# Vitamin D Association With Macrophage-Derived Cytokines in Polycystic Ovary Syndrome: An Enhanced Risk of COVID-19 Infection?

**DOI:** 10.3389/fendo.2021.638621

**Published:** 2021-02-25

**Authors:** Abu Saleh Md Moin, Thozhukat Sathyapalan, Alexandra E. Butler, Stephen L. Atkin

**Affiliations:** ^1^ Diabetes Research Center (DRC), Qatar Biomedical Research Institute (QBRI), Hamad Bin Khalifa University (HBKU), Qatar Foundation (QF), Doha, Qatar; ^2^ Academic Endocrinology, Diabetes and Metabolism, Hull York Medical School, Hull, United Kingdom; ^3^ Research Department, Royal College of Surgeons of Ireland, Manama, Bahrain

**Keywords:** COVID-19 risk factors, polycystic ovary disease, vitamin D, macrophage, cytokines

## Abstract

**Background:**

Women with polycystic ovary syndrome (PCOS) often have vitamin D deficiency, a known risk factor for severe COVID-19 disease. Alveolar macrophage-derived cytokines contribute to the inflammation underlying pulmonary disease in COVID-19. We sought to determine if basal macrophage activation, as a risk factor for COVID-19 infection, was present in PCOS and, if so, was further enhanced by vitamin D deficiency.

**Methods:**

A cross-sectional study in 99 PCOS and 68 control women who presented sequentially. Plasma levels of a macrophage-derived cytokine panel were determined by Slow Off-rate Modified Aptamer (SOMA)-scan plasma protein measurement. Vitamin D was measured by tandem mass spectroscopy.

**Results:**

Vitamin D was lower in PCOS women (p<0.0001) and correlated negatively with body mass index (BMI) in PCOS (r=0.28, p=0.0046). Basal macrophage activation markers CXCL5, CD163 and MMP9 were elevated, whilst protective CD200 was decreased (p<0.05); changes in these variables were related to, and fully accounted for, by BMI. PCOS and control women were then stratified according to vitamin D concentration. Vitamin D deficiency was associated with decreased CD80 and IFN-γ in PCOS and IL-12 in both groups (p<0.05). These factors, important in initiating and maintaining the immune response, were again accounted for by BMI.

**Conclusion:**

Basal macrophage activation was higher in PCOS with macrophage changes related with increased infection risk associating with vitamin D; all changes were BMI dependent, suggesting that obese PCOS with vitamin D deficiency may be at greater risk of more severe COVID-19 infection, but that it is obesity-related rather than an independent PCOS factor.

## Background

Polycystic ovary syndrome (PCOS) is considered to be a cardiometabolic condition with consequences that include obesity and insulin resistance that drive the excess prevalence of type 2 diabetes, hypertension, and cardiovascular diseases in later life (1). It has been suggested that these features of PCOS put subjects at a higher risk for severe COVID-19 infection (2, 3). Those with PCOS are more commonly affected by vitamin D deficiency than those without PCOS (4), deficiency occurring in over 60% of subjects. Controversially, vitamin D deficiency has been suggested to increase the risk and severity of COVID-19 disease, with an inverse correlation of COVID-19 incidence and mortality to vitamin D levels (5, 6); however, others have reported that there is no link between vitamin D and mortality (7).

In severe COVID-19 disease, acute respiratory distress syndrome (ARDS) results, caused by an unconstrained systemic inflammation to which differing populations of macrophages (resident alveolar macrophages (AMs), and recruited macrophages from the circulation) contribute (8). Macrophages are key players in inflammation and, upon activation, two polarized states result in an activated phenotype M1, macrophages that are pro-inflammatory and cytotoxic, and an activated phenotype M2, macrophages that are involved in tissue remodeling and matrix deposition (9, 10). Inflammation has been suggested to underlie insulin resistance and obesity in PCOS caused by macrophage stimulation (11); therefore, we hypothesized that there would be an increase in activated macrophages in those subjects with PCOS that would be further increased by vitamin D deficiency, predisposing these women to increased risk for severe COVID-19 disease.

## Methods

### Study Population

99 PCOS and 68 control women “who presented sequentially to the Department of Endocrinology, Hull and East Yorkshire Hospitals NHS Trust were recruited to the local PCOS biobank (ISRCTN70196169) from January 2014 to December 2016. The Newcastle & North Tyneside Ethics committee approved this study; all patients gave written informed consent (2).

PCOS diagnosis was based on the Rotterdam consensus diagnostic criteria, namely clinical or biochemical evidence of hyperandrogenism (Ferriman-Gallwey score >8; free androgen index >4.5 respectively), self-reported oligomenorrhea (≤ 9 menses per year) or amenorrhea (no menses for 3 months or more) and polycystic ovaries on transvaginal ultrasound (≥12 antral follicles in at least one ovary or ovarian volume of ≥10 cm^3^) (12). Study participants had no concurrent illness, were not on any medication for the preceding 9 months and were not planning to conceive. All PCOS women fulfilled the NIH criteria for diagnosis of PCOS (2).”

### Collection and Analysis of Blood Samples

Blood samples were collected and were measured in the Chemistry Laboratory, Hull Royal Infirmary, UK as previously described (13). “Insulin, C-reactive protein (CRP) and sex hormone binding globulin (SHBG) were measured by an immunometric assay with fluorescence detection on the DPC Immulite 2000 analyzer using the manufacturer’s recommended protocol, as previously described (13). Testosterone was measured by isotope dilution liquid chromatography-tandem mass spectrometry (Waters Corporation, Manchester, UK) as previously described (14).

The free androgen index (FAI) was calculated as the total testosterone x 100/SHBG. Serum insulin was assayed using a competitive chemiluminescent immunoassay performed on the manufacturer’s DPC Immulite 2000 analyzer (Euro/DPC, Llanberis, UK). The analytical sensitivity of the insulin assay was 2 μU/ml, the coefficient of variation was 6%, and there was no stated cross-reactivity with proinsulin. Plasma glucose was measured using a Synchron LX 20 analyzer (Beckman-Coulter), using the manufacturer’s recommended protocol. The coefficient of variation for the assay was 1.2% at a mean glucose value of 5.3 mmol/L during the study period. The insulin resistance was calculated using the HOMA method [HOMA-IR= (insulin x glucose)/22.5].” All analyses were undertaken according to current guidelines, regulations and quality control. Serum vitamin D levels and testosterone were quantified using isotope-dilution liquid chromatography tandem mass spectrometry (LC-MS/MS) (15): vitamin D sufficiency was defined as >70 ng/ml, insufficiency as 50–69 ng/ml and deficiency as <50 ng/ml (16).

### SOMA-Scan Assay

Plasma levels of macrophage-related proteins were determined by Slow Off-rate Modified Aptamer (SOMA)-scan plasma protein measurement as has been previously described (17, 18). The macrophage panel included measurement of M1 macrophage activation biomarkers (cytokines TNF-α, IL-6, IL-1β, IL-12, CD80 and chemokines CXCL1, CXCL2/CXCL3, CXCL5, CXCL8, CXCL9, CXCL10, CCL5, TLR4); activated M2 macrophage biomarkers (LBP, CD163, TFGβ-1, CD200, CD200R1, MMP7, MMP9, and CD36); conventional mediators of both M1 and M2 macrophage activation markers (IFN-γ, IL-4, IL-13). “The SOMAscan assay used to quantify proteins was performed on an in-house Tecan Freedom EVO liquid handling system (Tecan Group, Maennedorf, Switzerland) utilizing buffers and SOMAmers from the SOMAscan HTS Assay 1.3K plasma kit (SomaLogic, Boulder, CO) according to manufacturer’s instructions and as described previously (19, 20). The assay was performed in 96-well plates containing up to 85 plasma samples, three quality control and five calibrator plasma samples. Briefly, EDTA plasma samples were diluted into bins of 40%, 1%, and 0.05% and incubated with streptavidin-coated beads immobilized with dilution-specific SOMAmers *via* a photocleavable linker and biotin. After washing bound proteins were first biotinylated and then released from beads by photocleaving the SOMAmer-bead linker. The released SOMAmer-protein complex was treated with a polyanionic competitor to disrupt unspecific interactions and recaptured on the second set of streptavidin-coated beads. Thorough washing was performed before 5’ Cy3 fluorophore labelled SOMAmers were released under denaturing conditions, hybridized on microarray chips with SOMAmer-complementary sequences, and scanned using the SureScan G2565 Microarray Scanner (Agilent, Santa Clara, CA) (17).

#### Data Processing and Statistics

As previously described (17) “initial Relative Fluorescent Units (RFUs) were obtained from microarray intensity images using the Agilent Feature Extraction Software (Agilent, Santa Clara, CA). Raw RFUs were normalized and calibrated using the software pipeline provided by SomaLogic.” Comparisons were performed using Student’s t-test where a p-value <0.05 was taken as significant (GraphPad Prism 8.0, San Diego, CA, USA). No power analysis could be performed for this study because no data available relating the effect of vitamin D upon macrophage proteins in PCOS is available.

## Results

The PCOS women were older (p=0.03) with elevated BMI (p<0.001), weight (p<0.0001), waist and hip circumference (p<0.0001), systolic (p<0.001), and diastolic (p=0.03) blood pressure (**Table 1**). Biochemically, the PCOS women had elevated anti-Mullerian hormone (AMH) (p<0.0001), CRP (p<0.0001), testosterone (p=0.001) and free androgen index (p<0.0001). Vitamin D was significantly lower in the PCOS group (p<0.0001) and correlated negatively with BMI in PCOS (r=0.28, p=0.0046) (**Table 1**).

**Table 1:** Demographic and biochemical characteristics of the PCOS and control women. Data are presented as mean (SD).

	CONTROL (n = 68)	PCOS (n = 99)	P value
Age (years)	27.5 (0.6)	29.8 (0.9)	0.03
BMI (kg/m2)	26.6 (0.8)	34.6 (0.8)	<0.0001
Weight (kg)	73.7 (2.1)	97.8 (2.3)	<0.0001
Waist circumference (cm)	81 (2)	102 (2)	<0.0001
Hip circumference (cm)	101 (1)	119 (2)	<0.0001
Systolic blood pressure (mmHg)	115 (1)	122 (2)	0.0008
Diastolic blood pressure (mmHg)	74 (1)	77 (1)	0.03
AMH (pmol/l)	22.3 (2.1)	46.2 (3.2)	<0.0001
Sex Hormone Binding Globulin (SHBG) (nmol/l)	72.6 (9.5)	42.9 (4.5)	0.002
CRP (mmol/l)	2.1 (0.5)	4.8 (0.6)	<0.0001
Testosterone (nmol/l)	1.1 (0.1)	1.5 (0.1)	0.001
FAI	2.1 (0.2)	5.7 (0.6)	<0.0001
HOMA-IR	1.64 (1.60)	3.92 (6.22)	0.0052
Total vitamin D (ng/ml)	62 (3)	43 (3)	<0.0001

### Macrophage Proteins in PCOS

**Figure 1:**
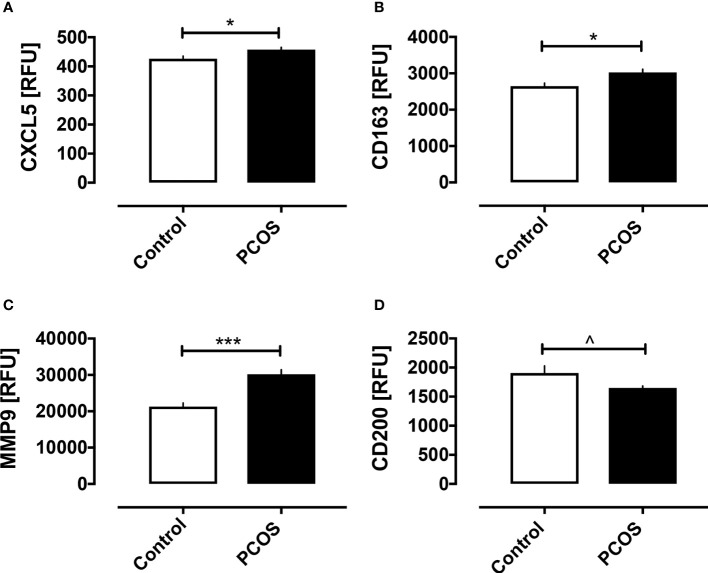
Macrophage-related proteins in women with and without PCOS. Baseline macrophage proteins are shown for the proteins that differed between PCOS and controls: CXCL5 **(A)**, CD163 **(B)**, MMP9 **(C),** and CD200 **(D)**. *p < 0.01, ***p < 0.0001, ^p < 0.05. RFU, relative fluorescent units.

**Figure 2:**
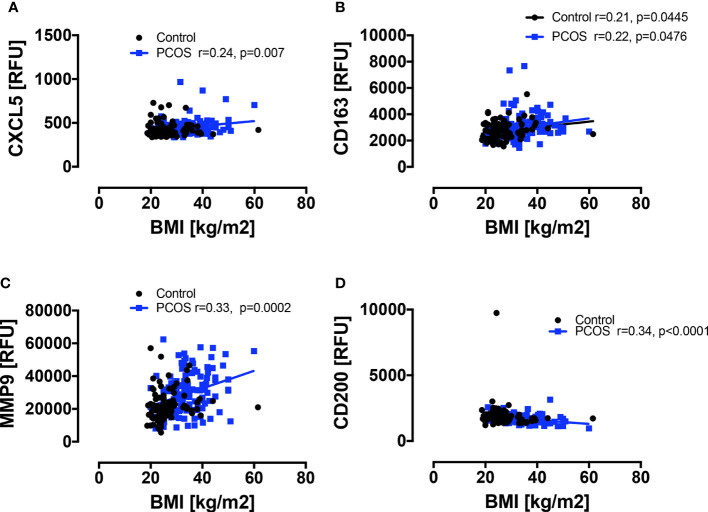
Correlations of macrophage-related proteins with BMI in PCOS and control women. A positive correlation with BMI was seen in PCOS women only for CXCL5 **(A)** and MMP9 **(C)**, and for CD163 **(B)** in both PCOS and control women; a negative correlation with BMI was seen in PCOS women only for CD200 **(D)**.

Baseline macrophage proteins are shown in **Figure 1**. Basal macrophage activation markers CXCL5, CD163 and MMP9 were elevated, whilst the protective CD200 was decreased (p<0.05); their correlation with BMI is shown in **Figure 2** and the changes in these variables were related to and fully accounted by BMI. The additional macrophage proteins that did not differ between controls and PCOS are shown in **Supplementary Figure 1** (10.6084/m9.figshare.13090652).

### Vitamin D Stratified Groups

**Figure 3:**
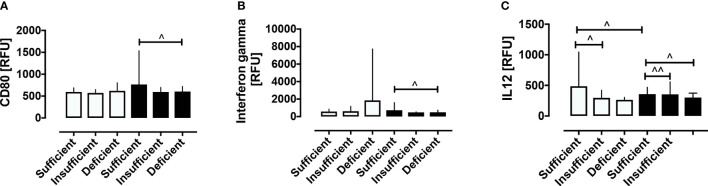
Stratification according to vitamin D status of macrophage-related proteins in women with and without PCOS. Stratification according to vitamin D status revealed that vitamin D deficiency was associated with decreased CD80 **(A)**, IFN-γ **(B)** in PCOS, and decreased IL-12 **(C)** in both PCOS and control women. ^p < 0.05, ^^p < 0.005. RFU, relative fluorescent units.

The PCOS and control women were then stratified according to vitamin D status. Vitamin D status was stratified into sufficient, insufficient and deficient. Of the 99 PCOS women, 16 (16%) were sufficient, 11 (11%) were insufficient and 72 (73%) were deficient. Of the 68 control women, 26 (38%) were sufficient, 22 (32%) insufficient, and 20 (29%) deficient (**Supplementary Table 1**). Vitamin D deficiency was associated with decreased CD80 and IFN-γ in PCOS (both p<0.05) and IL-12 (p<0.05) in both PCOS and controls, as shown in **Figure 3**. Those proteins that did not differ within groups stratified for vitamin D are shown in **Supplementary Figure 2** (10.6084/m9.figshare.13090652). As noted above, vitamin D correlated negatively with BMI in PCOS (r=0.28, p=0.0046) and the changes in CD80, IL-12 and, IFN-γ did not differ when BMI was adjusted for.

### BMI Stratified Groups

The macrophage-related proteins that were significantly different between PCOS and control groups were then divided into lean (BMI less than or equal to 25 kg/m^2^) and obese (BMI 26 kg/m^2^ or above).

In PCOS women, CD163 (p<0.005) and MMP9 (p<0.005) were elevated while CD200 (p<0.05) was reduced in the obese relative to the lean group. No difference was seen for CXCL5 between lean and obese PCOS women. There were no differences in these protein levels seen between lean and obese control women.

## Discussion

Macrophage-derived cytokines promote the inflammation in ARDS, and post-mortem lung histopathology in COVID-19 disease reveals inflammatory infiltrates of macrophages in the alveolar lumina (21). Systemic cytokine profiles of macrophage activation syndrome resemble that seen in patients with severe COVID-19 disease (22).

Low grade inflammation in PCOS is the key mediator of the insulin resistance and metabolic effects seen and are promoted by cytokines derived from macrophages (11). This study shows that basal macrophage-derived biomarkers associated with inflammation, CXCL5, CD163, and MMP9, were elevated in subjects with PCOS while CD200 was decreased. CXCL5 is a proinflammatory chemokine that promotes insulin resistance and is secreted from white adipose tissue in excess in obesity. CXCL5 correlated with BMI and, when the data were corrected for BMI, it was no longer significant, in accord with the serum levels reported to being no different in normal weight PCOS versus controls (23).

Soluble CD163 is a biomarker of macrophage activation that is associated with the development of diabetes (24). Elevated serum levels of CD163 have been reported in PCOS (25) though the mRNA levels in adipose tissue of PCOS and overweight individuals did not differ (26). This is in accord with the data reported here, where CD163 levels correlated with BMI and were no longer significant when the data were adjusted for BMI.

Matrix metalloproteinases (MMPs) are macrophage M2 markers that have been suggested to be important in the pathogenesis of PCOS, with reports differing with regard to serum MMP9 elevation or not (27). In this study, MMP9 was elevated basally in PCOS, in accord with previous reports (27); however, MMP9 correlated with BMI, and when the data were adjusted for BMI, significance was lost. Thus, this would explain the differing reports on MMP9 serum levels if BMI was not taken into account.

CD200 expression has been associated with a shift away from proinflammatory macrophages and therefore its reduction would promote the inflammatory process (28); this is in accord with our findings where basal levels of CD200 were reduced in PCOS women in this study. CD200 correlated with BMI and, when the data was adjusted for BMI, then CD200 was no longer significantly reduced in PCOS.

Overall, it can be seen that the proinflammatory expression of macrophage-derived proteins seen in PCOS were all driven by obesity and were therefore not independent markers of inflammation in PCOS.

Vitamin D deficiency has been shown to be related to the expression of proinflammatory macrophage cytokines and fibrosis (29), factors that may contribute to its association with a poor outcome in COVID-19 disease (5, 6). The data here show that vitamin D deficiency was associated with decreased CD80 and IFN-γ in PCOS (both p<0.05), and IL-12 (p<0.05) in both PCOS and controls. CD80 is a costimulatory molecule produced by macrophages that is important in maintaining T cell activation (30), while IL-12 activates natural killer and cytotoxic T lymphocytes that are important as mediators of inflammation-induced apoptosis (31). However, in all cases, after adjustment for BMI, none of these proteins remained significantly altered. This suggests that if vitamin D deficiency is a risk factor for increased severity of COVID-19 disease in PCOS, then the mechanism is not through macrophage cytokine mediation independent of obesity.

After ingestion or production in the skin, the fat-soluble prohormone vitamin D affects many physiological functions, including the regulation of both innate and adaptive immunity (32–34). Activation of vitamin D can occur through canonical and non-canonical pathways. In the classic pathway, vitamin D is first metabolized to 25-hydroxyvitamin D_3_ by CYP2R1 and CYP27A1 in the liver, then in the kidney and other organs, such as the skin and the immune system, to the active 1,25-dihydroxyvitamin D_3_ by CYP27B1 (35–37). In the alternative pathway, vitamin D is activated by CYP11A1, resulting in the production of over 10 different metabolites (36, 38–43); this includes activation of lumisterol, a photoproduct of pro-vitamin D (44).

Both 1,25-dihydroxyvitamin D_3_ and the CYP11A1-derived metabolites can affect immune functions (39), and much evidence supports their potent anti-inflammatory and antioxidative activities (45). While the possible association between vitamin D deficiency/insufficiency and more severe COVID-19 disease remains speculative, there is increasing evidence in support of the potential role of classical and alternative forms of vitamin D in damping down the production of pro-inflammatory cytokines (cytokine storm) and oxidative stress induced by COVID-19 infection and thus mitigating their harmful effects (45).

Limitations of this study include that it was only a moderately sized cross-sectional study and that only total vitamin D was measured and not the active 1,25 dihydroxyvitamin D or its metabolites that may also be active. In addition, no functional assays were undertaken, and only circulatory levels of macrophage-related proteins were measured that may not reflect concentrations at the tissue level.

## Conclusions

In conclusion, obese subjects with PCOS show a basal proinflammatory macrophage-derived protein profile together with vitamin D deficiency that was also associated with reduced T cell regulatory proteins compared to controls. However, all of these features could be accounted for by BMI suggesting that obese, but not lean, PCOS subjects may be at risk for more severe COVID-19 disease and that vitamin D effects on macrophage-related proteins is not independent of obesity.

## Data Availability Statement**

The raw data supporting the conclusions of this article will be made available by the authors, without undue reservation.

## Ethics Statement**

The studies involving human participants were reviewed and approved by The Newcastle & North Tyneside Ethics committee. The patients/participants provided their written informed consent to participate in this study.

## Author Contributions

AM and AB analyzed the data and wrote the manuscript. TS supervised clinical studies and edited the manuscript. SA contributed to study design, data interpretation and the writing of the manuscript. All authors contributed to the article and approved the submitted version. AB is the guarantor of this work.

## Conflict of Interest

The authors declare that the research was conducted in the absence of any commercial or financial relationships that could be construed as a potential conflict of interest.

## Supplementary Material

Supplementary Figure 1 Macrophage-related proteins where no difference was seen between PCOS and control women.

Supplementary Figure 2 Macrophage-related proteins stratified according to vitamin D status where no difference was seen between stratified groups for either PCOS or control women.

## Abbreviations

PCOS, polycystic ovary syndrome; SOMA, Slow Off-rate Modified Aptamer; RFU, Relative Fluorescent Units; CRP, C-reactive protein; SHBG, sex hormone binding globulin; BMI, body mass index; LC-MS/MS, liquid chromatography tandem mass spectrometry; ARDS, acute respiratory distress syndrome; AM, alveolar macrophages; COVID-19, coronavirus disease of 2019; MMPs, Matrix metalloproteinases; TNF-α, Tumor Necrosis Factor alpha; IL-6, Interleukin-6; IL-1β, Interleukin-1 beta; IL-12, Interleukin-12; CD80, Cluster of differentiation 80; CXCL1, chemokine (C-X-C motif) ligand 1; CXCL2/CXCL3, chemokine (C-X-C motif) ligand 2/chemokine (C-X-C motif) ligand 3; CXCL5, chemokine (C-X-C motif) ligand 5; CXCL8, chemokine (C-X-C motif) ligand 8; CXCL9, chemokine (C-X-C motif) ligand 9; CXCL10, chemokine (C-X-C motif) ligand 10; CCL5, Chemokine (C-C motif) ligand 5; TLR4, Toll-like receptor 4; LBP, Lipopolysaccharide-binding protein; CD163, Cluster of Differentiation 163; TFGβ-1, transforming growth factor-β1; CD200, Cluster of Differentiation 200; CD200R1, Cluster of Differentiation 200 receptor 1; MMP7, Matrix metalloproteinase-7; MMP9, Matrix metalloproteinase-9; CD36, Cluster of Differentiation 36; IFN-γ, Interferon gamma; IL-4, Interleukin-4; IL-13, Interleukin-13.
