# Supplementary figures and images for: Vitamin D Association With Macrophage-Derived Cytokines in Polycystic Ovary Syndrome: An Enhanced Risk of COVID-19 Infection?
Source: Front Endocrinol (Lausanne). 2021 Feb 25;12:638621. doi: 10.3389/fendo.2021.638621 (PMC7947877; doi:10.3389/fendo.2021.638621)

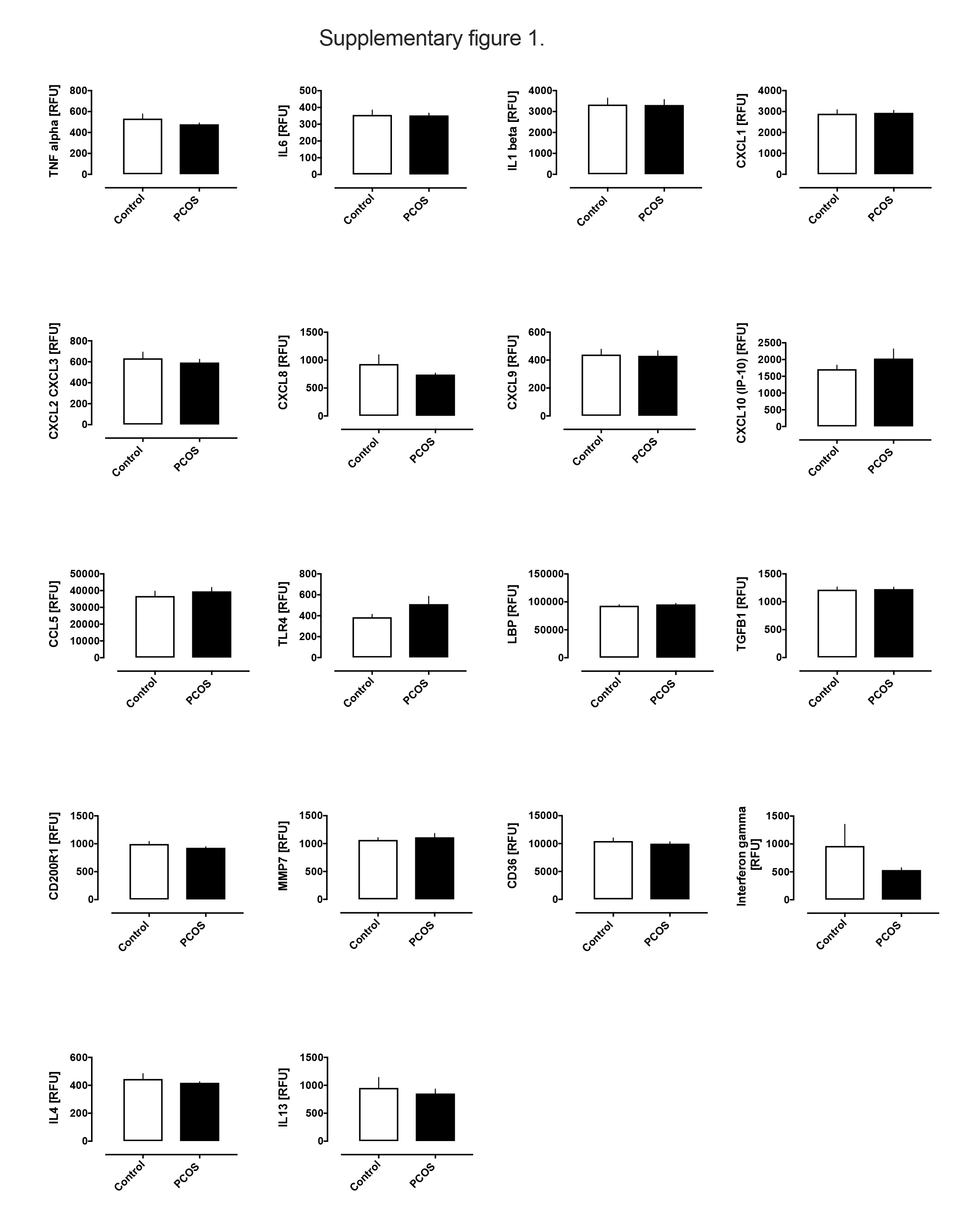

Supplement: Supplementary Figure 1 — Macrophage-related proteins where no difference was seen between PCOS and control women. [file Image_1.tif]

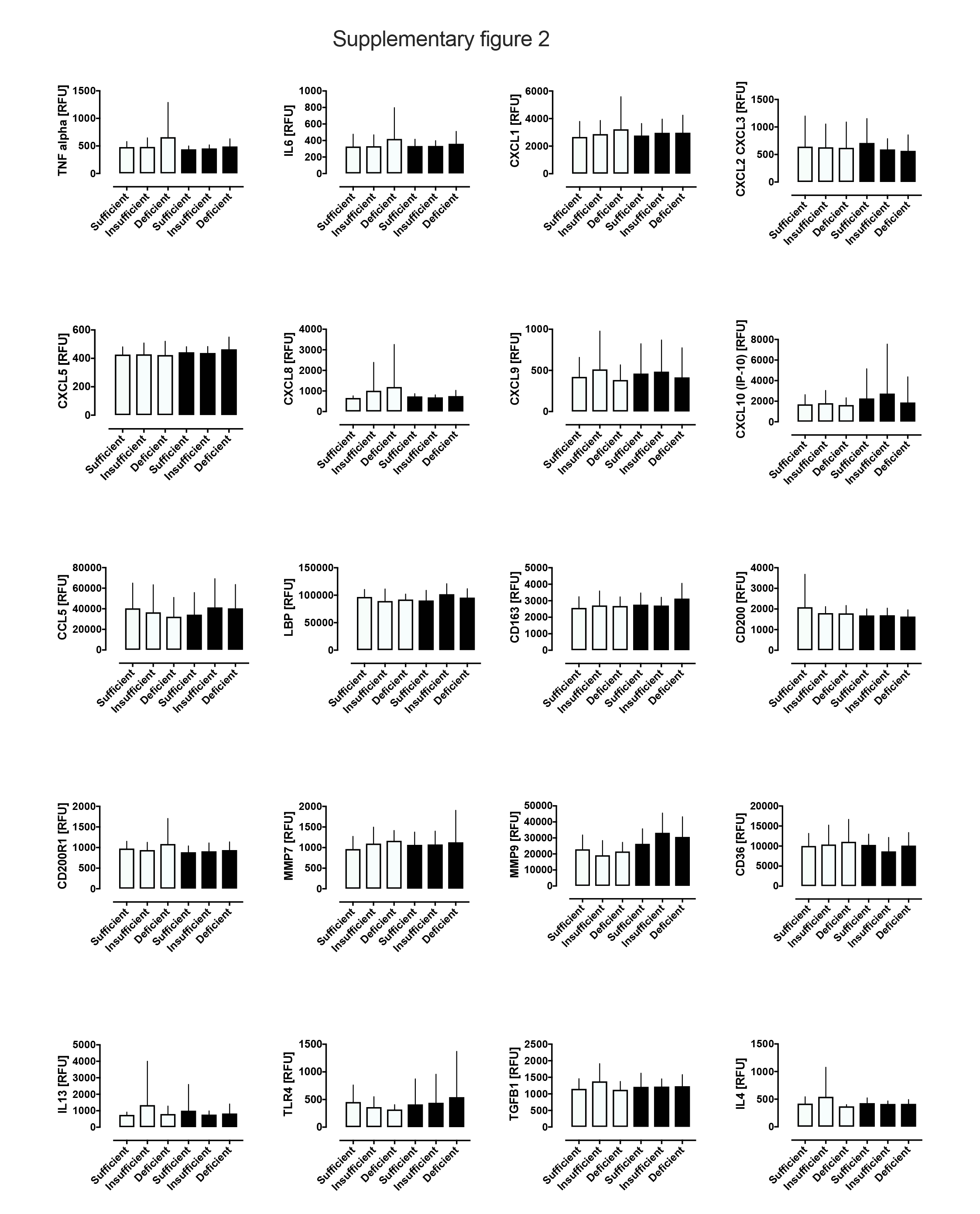

Supplement: Supplementary Figure 2 — Macrophage-related proteins stratified according to vitamin D status where no difference was seen between stratified groups for either PCOS or control women. [file Image_2.tif]
