# Supplementary material for: Vitamin D Association With Macrophage-Derived Cytokines in Polycystic Ovary Syndrome: An Enhanced Risk of COVID-19 Infection?
Source: Front Endocrinol (Lausanne). 2021 Feb 25;12:638621. doi: 10.3389/fendo.2021.638621 (PMC7947877; doi:10.3389/fendo.2021.638621)
Supplement: Supplementary file 3 [file Table_1.docx]

**Supplementary Table 1.** Demographic and biochemical characteristics of the PCOS and control women, stratified according to vitamin D status. Data are presented as mean (SD).

|  |  |  |  |  |  |  |  |
| --- | --- | --- | --- | --- | --- | --- | --- |
|  |  | **CONTROL (n=68)** |  |  | **PCOS (n=99)** |  |  |
|  | **Vitamin D sufficient (n=26) (38%)** | **Vitamin D insufficient (n=22) (32%)** | **Vitamin D deficient (n=20) (29%)** | **Vitamin D sufficient (n=16) (16%)** | **Vitamin D insufficient (n=11) (11%)** | **Vitamin D deficient (n=72)**  **(73%)** |  |
| Age (years) | 29.4 (7.0) | 29.0 (6.3) | 30.7 (7.2) | 29.1 (6.2) | 28.5 (6.0) | 26.8 (6.1) |  |
| BMI (kg/m2) | 25.2 (3.7) | 28.0 (9.2) | 26.7 (5.2) | 31.1 (5.0) | 32.8 (7.4) | 35.6 (7.7) |  |
| Weight (kg) | 71.9 (11.8) | 75.6 (24.8) | 74.1 (14.4) | 86.4 (17.7) | 91.5 (20.6) | 101.4 (23.7) |  |
| Waist circumference (cm) | 79.6 (8.8) | 81.3 (15.8) | 83.5 (13.0) | 93.6 (12.2) | 101.9 (18.7) | 103.4 (16.1) |  |
| Hip circumference (cm) | 99.7 (10.3) | 100.0 (11.5) | 102.6 (11.6) | 110.7 (13.2) | 117.2 (14.7) | 120.7 (16.7) |  |
| Systolic blood pressure (mmHg) | 113.6 (10.3) | 115.0 (15.5 | 116.7 (8.4) | 118.9 (12.1) | 119.0 (17.8) | 123.6 (14.5) |  |
| Diastolic blood pressure (mmHg) | 72.2 (12.6) | 73.1 (12.4) | 75.8 (9.1) | 78.5 (11.6) | 74.0 (12.2) | 77.7 (10.3) |  |
| AMH (pmol/l) | 20.1 (13.4) | 26.8 (21.6) | 20.2 (16.3) | 44.7 (42.6) | 36.8 (22.3) | 47.8 (30.3) |  |
| SHBG (nmol/l) | 84.8 (101.5) | 64.6 (48.5) | 65.7 (62.2) | 38.6 (23.4) | 40.3 (38.7) | 44.3 (47.9) |  |
| Cholesterol (mm/l) | 4.7 (0.9) | 4.6 (0.6) | 4.7 (0.7) | 5.2 (0.3) | 4.5 (0.5) | 4.8 (1.0) |  |
| Triglycerides (mmol/l) | 1.1 (0.8) | 1.0 (0.9) | 1.0 (0.8) | 1.3 (0.7) | 1.3 (0.6) | 1.7 (1.6) |  |
| HDL (mmol/l) | 1.5 (0.4) | 1.5 (0.3) | 1,3 (0.3) | 1.2 (0.3) | 1.2 (0.3) | 1.2 (0.3) |  |
| LDL (mmol/l) | 2.7 (0.8) | 2.8 (0.6) | 2.8 (0.5) | 3.4 (1.2) | 2.6 (0.8) | 2.8 (0.9) |  |
| CRP (mmol/l) | 2.8 (5.0) | 2.2 (4.3) | 1.7 (1.7) | 3.6 (2.3) | 4.9 (3.6) | 5.1 (4.8) |  |
| Testosterone (nmol/l) | 1.0 (0.4) | 1.1 (0.6) | 1.1 (0.6) | 1.0 (0.4) | 1.5 (1.1) | 1.5 (0.8) |  |
| Androstenedione (nmol/l) | 8.1 (3.5) | 8.9 (7.3) | 7.3 (3.6) | 11.2 (5.9) | 9.1 (4.9) | 11.0 (5.5) |  |
| FAI | 2.1 (1.2) | 2.1 (1.3) | 2.2 (1.3) | 3.4 (1.9) | 5.5 (5.8) | 6.3 (6.0) |  |
| Glucose (mmol/l) | 4.5 (0.3) | 5.1 (1.2) | 4.5 (0.5) | 4.7 (0.2) | 4.9 (0.5) | 4.9 (1.3) |  |
| Insulin (μU/ml) | 5.8 (3.8) | 9.4 (8.1) | 6.8 (3.4) | 10.9 (5.2) | 11.9 (9.5) | 19.1 (16.2) |  |
| HOMA-IR | 1.20 (0.16) | 2.35 (0.53) | 1.41 (0.17) | 2.29 (0.31) | 2.56 (0.66) | 4.52 (0.92) |  |
| White cell count (x10^9^/l) | 5.8 (1.3) | 5.7 (1.5) | 5.3 (1.1) | 6.6 (1.5) | 7.6 (2.1) | 7.1 (1.9) |  |
| Platelets (x10^9^/l) | 257 (59) | 254 (48) | 250 (42) | 286 (54) | 284 (46) | 274 (61) |  |
| Total vitamin D (ng/ml) | 85.1 (12.4) | 61.0 (6.3) | 33.1 (12.6) | 88.9 (17.8) | 59.3 (5.2) | 29.1 (12.3) |  |
|  |  |  |  |  |  |  |  |

SHBG: Sex Hormone Binding Globulin
